# Socioeconomic inequalities in healthcare system efficiency in Japan during COVID-19 pandemic: an analysis of the moderating role of vaccination

**DOI:** 10.3389/fpubh.2024.1170628

**Published:** 2024-03-21

**Authors:** Yin Tang

**Affiliations:** Graduate School of Economics, Keio University, Tokyo, Japan

**Keywords:** healthcare system efficiency, socioeconomic inequality, COVID-19, vaccination, moderating effect, Japan

## Abstract

**Background:**

In the context of the COVID-19 pandemic, limited research has focused on socioeconomic disparities in Local Healthcare System Efficiency (LHSE) among Japanese prefectures. This study seeks to investigate the moderating impact of vaccination on the relationship between LHSE and socioeconomic characteristics and endowments

**Methods:**

To explore these relationships, we first utilized the Data Envelopment Analysis with Slack-Based Measure to measure the LHSE, based on data from Japanese prefectures during waves 2 to 5 of the pandemic. Then estimating the impact of socioeconomic variables on LHSE. Finally, we assessed the changes in the way socioeconomic variables affect LHSE before and after vaccine deployment using the Seemingly Unrelated Estimation t-test methodology.

**Results:**

The research findings suggest an overall reduction in LHSE disparities across various regions due to the utilization of vaccines. Particularly in areas with relatively nsufficient bed resources, a significant improvement in LHSE was observed in most regions. However, there was no evidence supporting the role of vaccine deployment in mitigating socioeconomic inequalities in LHSE. Conversely, the utilization of vaccines showed a positive correlation between the improvement in LHSE and the proportion of older adult population in regions with sufficient bed resources. In regions facing bed shortages, the enhancement of LHSE became more reliant on reducing the occupancy rate of secured beds for severe cases after the introduction of vaccination.

**Discussion:**

In regions facing bed shortages, the enhancement of LHSE became more reliant on reducing the occupancy rate of secured beds for severe cases. This underscores the importance for policymakers and implementers to prioritize the treatment of severe cases and ensure an effective supply of medical resources, particularly secured beds for severe cases, in their efforts to improve LHSE, in the post-COVID-19 era with rising vaccine coverage.

## 1 Introduction

Approximately four years after the World Health Organization declared coronavirus disease 2019 (COVID-19) a Public Health Emergency of International Concern on January 30, 2020, Japan continues to grapple with the pandemic. The Japanese paradox, denoting limited fatalities despite relaxed restrictions (1, 2), has garnered global attention in the fight against COVID-19. Despite Japan’s avoidance of strict lockdowns, which are prevalent in many parts of Europe and the United States, it remarkably maintains the lowest mortality rate among all OECD countries (3). In addition to the distinctive lifestyle habits of the Japanese (4) and effective government policies for disease prevention (5–7), some studies attribute this phenomenon to the efficient allocation and coordination of medical resources by local authorities (1, 2, 8).

During the pandemic, almost all Japanese prefectures faced challenges due to insufficient medical resources and healthcare system disruptions. Prefectures can request cooperation from hospitals, and while this request is essentially a directive for public hospitals, it remains a request for private hospitals. It is important to note that the majority of healthcare institutions in Japan are privately operated, constituting approximately 80% of the total facilities. Shin, Takada (9) found that in April and May of 2020, hospitals in Japan experienced reduced hospital charges compared to the same period in 2019. Notably, for hospitals admitting COVID-19 patients, longer hospitalization periods for these patients, including suspected cases, led to greater reductions in hospital charges. The average additional cost reduction per COVID-19 patient was estimated at 5.5 million Japanese yen. Therefore, from a hospital management perspective, admitting COVID-19 patients may not be financially advantageous.

To enhance the healthcare system’s resilience to shocks and crises, it is crucial for the government to invest in the core functions of the healthcare system. Funding for public health services, encompassing infection prevention, control, surveillance, and information systems, is considered fundamental for ensuring preparedness and response to health emergencies (10). Various policies have been implemented to secure hospital resources and improve the healthcare delivery system since April 2020. Two key initiatives have been introduced as economic support measures: an additional hospital charge for severely ill COVID-19 patients and the provision of subsidies (referred to as the “COVID-19 Emergency Comprehensive Support Grant – Medical Portion” or ECSG) for healthcare system development projects established by each prefecture (11). The ECSG, established in the First Supplementary Budget for the fiscal year 2020, is intended for prefectures and is designed to support expenses related to projects conducted by prefectures, municipalities, private organizations, and others deemed appropriate by the prefecture (12). Therefore, the ECSG has given local governments discretionary authority to undertake urgently needed measures related to the response to COVID-19 to some extent.

In this study, local governments receive transfer payments, referred to as ECSG, from the central government to bolster and invest in the local healthcare system, aiming to mitigate the loss of human resources caused directly or indirectly by the COVID-19 pandemic. This input–output ratio is defined as the Local Healthcare System Efficiency (LHSE) and is estimated using the Data Envelopment Analysis (DEA) with Slack-Based Measure (SBM). Additionally, in the context of DEA, the Decision-Making Units (DMUs) refer to the prefectures in this study.

The primary objective of this study is to investigate the moderating effect of vaccine usage on the socioeconomic inequalities in LHSE. The moderating effect refers to the influence of a third variable on the relationship between the first and second variables (13). Then in the other word, this research is to examine the impact of vaccine introduction on the relationship between socioeconomic factors and LHSE. The motivation for this study arises from two key observations: firstly, during the COVID-19 pandemic, improvements in the LHSE exhibited socioeconomic inequality (14–16). For example, Lupu and Tiganasu (15) found that during the initial wave of the pandemic in Europe, factors like population density and the older adult population negatively impacted LHSE improvement. Secondly, the COVID-19 vaccination proves to have reduced the inequality in mortality (17, 18) and infection (19). The question at hand is whether the deployment of vaccines can reduce these socioeconomic inequalities in LHSE. A straightforward intuition is that vaccines, by increasing the recovery rate of COVID-19 patients per unit input, thus reducing the pressure on healthcare resources faced by DMUs with relatively unfavorable socioeconomic characteristics and endowments, may decrease the dependence of LHSE on socioeconomic factors. However, on the other hand, if DMUs do not timely adjust their healthcare system operational strategies post-vaccine deployment, there is a possibility of increased resource wastage, leading to a tighter connection between certain socioeconomic factors and LHSE.

The necessity of studying healthcare efficiency arises from the rapid changes in healthcare service supply and demand, especially in the context of limited fiscal resources (20). Japan is one of the most rapidly aging countries globally, and it boasts a relatively efficient and comprehensive healthcare system. However, during the early stages of the pandemic, most prefectures experienced significant healthcare system disruptions due to the surge in cases, leading them to implement non-medical measures to control the virus’s spread while ensuring the availability of healthcare resources, such as hospital beds and healthcare personnel (11). The introduction of vaccines is expected to bring regions closer to herd immunity by increasing antibody coverage, thereby reducing the pressure on both healthcare demand and supply. This alleviates the situation of supply shortages and eases the obstacles to healthcare system efficiency improvement posed by socioeconomic factors. However, as of mid-2023, there is still little research that can confirm this prediction.

This research investigates whether the vaccination reduces the disparity in the difficulty of enhancing healthcare efficiency between regions with different healthcare demand environments. The results indicate that the introduction of vaccines has, on the one hand, reduced socioeconomic inequalities on most aspects of LHSE. On the other hand, it has amplified the impact of severe cases on LHSE, particularly in prefectures with limited bed resources. This suggests that while the use of vaccines indeed lowers the difficulty for prefectures with different healthcare demand environments to improve LHSE, it also poses greater obstacles to regions with a higher prevalence of severe cases. These findings remind policymakers that, following improvements in healthcare supply technology led by vaccines, efforts to enhance LHSE should particularly focus on the prevention of severe cases and the treatment of critically ill patients.

This study examined whether vaccine administration could reduce the disparities in improving healthcare efficiency among regions with different healthcare demand environments. The results indicate that, on one hand, the introduction of vaccines did indeed overall reduce the regional disparities in LHSE. On the other hand, there’s no evidence to support that vaccine introduction moderated socioeconomic inequalities in LHSE. Instead, the introduction of vaccines intensified the negative impact of high occupancy rates of secured beds for severe cases on LHSE improvements in regions with insufficient bed resources. It also established a positive correlation between the proportion of older adult individuals in the population and LHSE improvements in regions with sufficient bed resources during the initial phase of vaccine deployment. These findings prompt policymakers that in the post-pandemic era with continuously expanding vaccine coverage, efforts to enhance LHSE should particularly focus on ensuring the supply of medical resources required for the treatment of severe cases.

This study makes two significant contributions: first, it focuses on LHSE in Japan and provides a visual representation of LHSE spatial distribution during different stages of the COVID-19 pandemic. Second, it goes beyond the perspective in existing literature that mainly considers mortality rates and infection rates as outcome indicators, by studying the impact of vaccination on socioeconomic inequality within LHSE for the first time.

The remainder of this study is structured as follows: Section 2 gives a brief introduction to prior studies on the assessment of LHSE and the effects of COVID-19 vaccination; Section 3 and Section 4 describe the research design and methodology, respectively, providing a detailed description of model construction, variable selection, and so on. Section 5 presents the results of the empirical analysis, explains their implications, and discusses the limitations of this study. Finally, Section 6 draws conclusions.

## 2 Materials and methods

**Figure 1:**
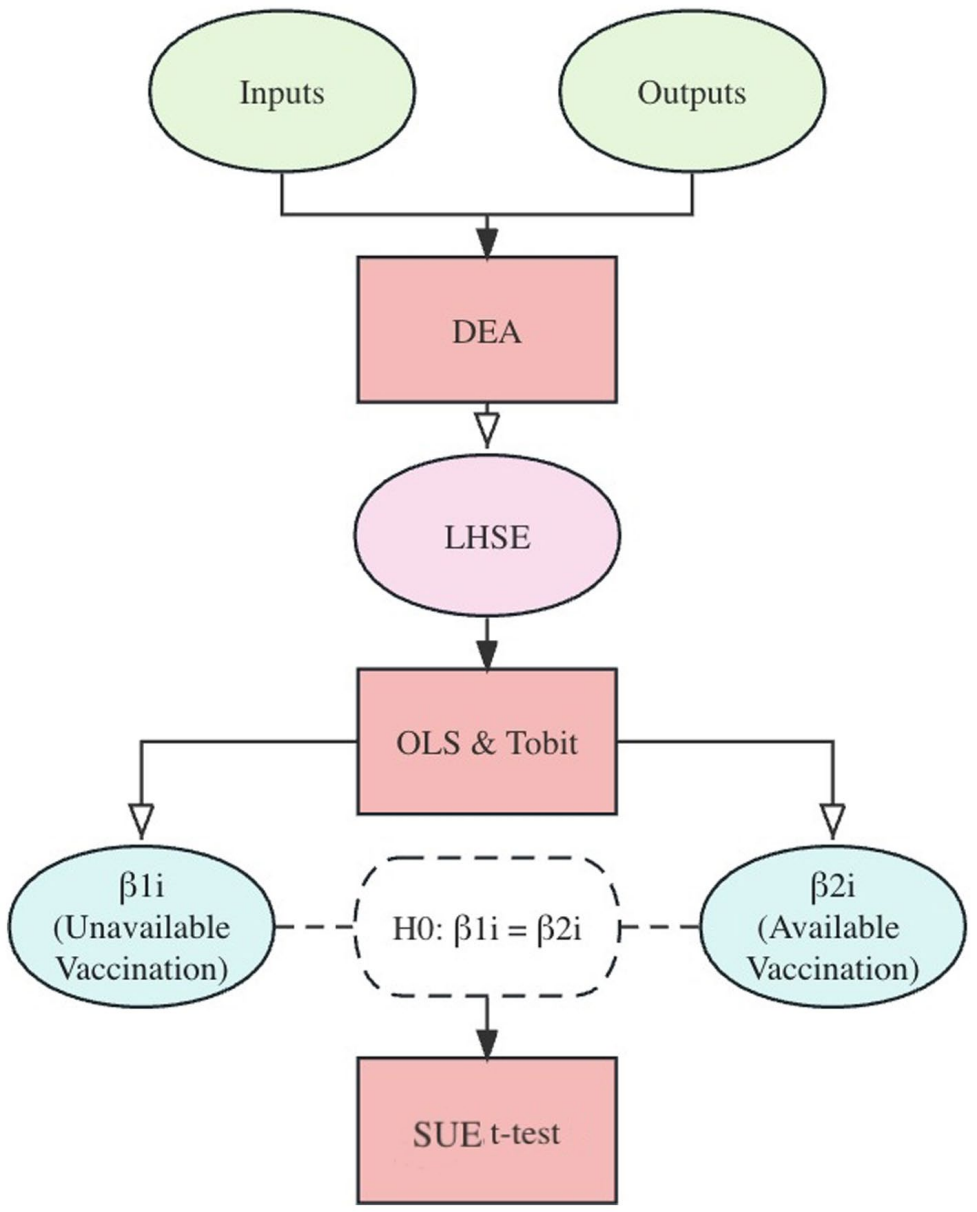
Research flow.

This study aims to investigate the changes in socioeconomic inequalities in LHSE before and after the administration of COVID-19 vaccines in Japan’s prefectures. To accomplish this objective, the research design process, as depicted in Figure 1, comprises (i) a cross-sectional evaluation of LHSE across prefectures for each wave using the SBM-DEA method, (ii) the identification of socioeconomic determinants of LHSE in each prefecture through the OLS and Tobit models, and (iii) the utilization of the Seemingly Unrelated Estimation (SUE) *t*-test to explore the moderating influence of vaccination on LHSE. The SBM-DEA is performed by MaxDEA X, and the OLS, Tobit regression, and the SUE *t*-test are carried out using Stata 16.

It is essential to clarify that this study concentrates on the healthcare efficiency of COVID-19. Consequently, economic consequences stemming from the pandemic, such as economic downturns and increases in unemployment, are not taken into account. Additionally, the study’s focus is on efficiency itself, rather than proposing methods to enhance it.

With reference to Paxson and Shen’s (21) research, I define an epidemic wave as the temporal evolution of dead persons with an isolated peak and tails. The study period spans from the second wave to the fifth wave of the COVID-19 pandemic in Japan, from August 1, 2020, to December 31, 2021. As of April 2023, Japan has experienced eight significant COVID-19 outbreaks, each associated with a notable surge in new infections and fatalities. To ensure consistency, the study excludes the initial outbreak, considering that different regions were affected at varying times during the first outbreak. For instance, the first confirmed COVID-19 case in Japan was reported on January 16, 2020, while no confirmed cases were recorded in Iwate prefecture as of August 7, 2020 (6).

Furthermore, according to the Japan Variant Report (22, 23), the Omicron variant became dominant in Japan in early 2022, replacing the Delta variant. Several studies suggested that the Omicron variant had high transmission (24, 25) but low pathogenicity (26–28) compared to previous variants. This led to the Japanese government and local authorities adopting a coexistence strategy with COVID-19 and gradually easing containment measures (29, 30). Consequently, the situation post-2022 significantly differs from the preceding circumstances. Therefore, data analysis is based exclusively on data up to December 31, 2021.

Due to the absence of official definitions for the start and end times of each pandemic wave in Japan, this study defines each wave of major COVID-19 outbreaks based on peaks and troughs in the trend of new deaths in the COVID-19 pandemic, and specific definitions are provided in Table 1. To simplify the analysis, the beginning and end of each wave are set on the first and last day of a month, respectively.

**Table 1:** Time periods of each wave.

Wave	Time period	Availability of vaccination
Wave 2	August 1, 2020–October 31, 2020	Unavailable
Wave 3	November 1, 2020–March 31, 2021	Unavailable
Wave 4	April 1, 2021–July 31, 2021	Available
Wave 5	August 1, 2021–December 31, 2021	Available

It is important to note that COVID-19 vaccination became available in Japan on February 14, 2021. Initially, the vaccine was authorized only for use by healthcare workers and the older adult due to limited availability. According to statistics from the Prime Minister’s Office of Japan (31), the count of fully vaccinated individuals began on May 3, 2021, during the fourth wave of the pandemic.

### 2.1 Estimation of local healthcare system efficiency scores

#### 2.1.1 Data enveloped analysis

The foundation of the DEA method dates back to Farrell (32). Building upon Farrell’s work, Charnes, Cooper (33) introduced the DEA with constant returns to scale (CRS), known as the CCR model. Banker, Charnes (34) extended the DEA to accommodate variable returns to scale (VRS), called the BCC model. Tone (35) proposed the classic SBM model as an improvement upon the traditional DEA model. This model incorporates slack variables to enhance the assessment of input–output relationships, allowing for non-proportional measures in different inputs or outputs based on distinct proportions.

In this section, I employ the SBM-DEA model with CRS assumption to estimate the efficiency scores of healthcare systems, because the CRS assumption aligns with the goal of evaluating efficiency rather than resource management or cost control within healthcare units, which is consistent with the purpose of DEA in this study (36). In SBM-DEA model, efficient DMUs are assigned a value of 1, while inefficient DMUs receive scores between 0 and 1.

Assuming there are *n* DMUs to be evaluated with *m* input indicators and *q* output indicators, we can determine the LHSE score of $DMU_{0}$ , denoted as $\theta_{0}$ , using the following Equation 1 with some constraints:

$$\theta_{0}=\min\frac{1-\frac{1}{m}\sum_{i=1}^{m}\frac{s_{i}^{-}}{x_{i0}}}{1+\frac{1}{q}\sum_{r=1}^{q}\frac{s_{r}^{+}}{y_{r0}}}$$

$$s.t.\overset{n}{\underset{j=1,\neq 0}{\sum }}x_{ij}\lambda_{j}+s_{i}^{-}=x_{i0}$$

(1) $$\overset{n}{\underset{j=1,\neq 0}{\sum }}y_{rj}\lambda_{j}-s_{r}^{+}=y_{r0}$$

$$\lambda_{j},s_{i}^{-},s_{r}^{+}\geq 0$$

i=1,…,m

r=1,…,q

j=1,…,n,

where $x_{ij}$ and $y_{rj}$ indicate the inputs and outputs of $DMU_{j}$ , respectively; $\theta_{0}$ is the target value; $s_{i}^{-}$ and $s_{r}^{+}$ are the slack variable and the residual variable, indicating the amount of inputs that need to be reduced and the amount of outputs that need to be increased to reach the optimal allocation, respectively.

#### 2.1.2 Indicators of input and output

This study employs several input indicators, including the primary projects of the ECSG, the number of PCR tests conducted, the number of COVID-19 vaccine shots administered, and the proportion of days the state of emergency declaration (DSE) was in effect relative to the total days of each pandemic wave. The primary activities of the ECSG encompass the projects for priority medical institution system development, hospital bed securing project and accommodation treatment facility securing, equipment improvement project of inpatient medical institution and priority medical, and facilities improvement project for returnees/contact persons outpatient facilities. Moreover, a cure rate is defined as the ratio of the number of new discharges or releases from isolation to the number of newly positive cases, serving as an output indicator. The specific definitions of each indicator are provided in Table 2.

**Table 2:** Notation and definition of inputs and outputs.

Indicator	Definition
**Inputs**	
ECSG_MIS	ECSG for priority medical institution system development project (Unit: thousand yen per 1,000,000 population)
ECSG_BA	ECSG for hospital bed securing project and accommodation treatment facility securing project (Unit: thousand yen per 1,000,000 population)
ECSG_FD	ECSG for equipment improvement project of inpatient medical institution and priority medical institution (Unit: thousand yen per 1,000,000 population)
ECSG_RCP	ECSG for facilities improvement project for returnees/contact persons outpatient facilities, etc. (Unit: thousand yen per 1,000,000 population)
PCR	Number of PCR tests in the period (Unit: cases per 1,000,000 population)
Vaccination	Number of COVID-19 vaccine doses^a^ in the period (Unit: cases per 100 population)
DSE	$DSE=\frac{\mathrm{Number}\;\mathrm{of}\;\mathrm{days}\;\mathrm{the}\;DSE\;\mathrm{was}\;\mathrm{in}\;\mathrm{force}}{\mathrm{Total}\;\mathrm{number}\;\mathrm{of}\;\mathrm{days}}$
**Output**	
Cure rate	$\mathrm{Cure}\;\mathrm{rate}=\frac{\mathrm{Number}\;\mathrm{of}\;\mathrm{the}\;\mathrm{cases}\;\mathrm{discharged}\;\mathrm{from}\;\mathrm{hospital}\;\mathrm{or}\;\mathrm{released}\;\mathrm{from}\;\mathrm{treatment}}{\mathrm{Number}\;\mathrm{of}\;\mathrm{the}\;\mathrm{newly}\;\mathrm{confirmed}\;\mathrm{cases}\;\mathrm{with}\;a\;10-\mathrm{day}\;\mathrm{lag}}$

a All does are counted except for the first does, considering that two doses of any WHO Emergency Use Listing vaccine to be a complete primary series.

The number of vaccine doses administered is based on data published by the Prime Minister of Japan and His Cabinet, while all other variables are derived from data provided by the Ministry of Health, Labour and Welfare of Japan.

It’s important to note that the ECSG covers the primary labor-related, capital-related, and consumable resources-related inputs, which are considered as three main input categories in the framework of the DEA assessing the efficiency of primary care and healthcare organizations (36). According to the Guidelines for the Fiscal Year 2020 COVID-19 Emergency Comprehensive Support Grant (Medical Portion) (12), all pertaining to activities aimed at preventing the spread of infection and enhancing the medical infrastructure are eligible for payment under the ECSG, such as the expenses related to staff wages, compensation, rewards, and the purchase of supplies and necessary expenses.

In addition to the above-mentioned input indicators, I argue that the influence of quarantine policy and *ad hoc* measures should be considered. NHK of Japan (37) reports that from April 7 to April 16, 2020 (the end of the first wave), the first DSE was issued throughout Japan, requesting residents of each prefecture to reduce their outings and gatherings, issuing restrictions on the operation of public facilities and places where people gather, and deploying medical resources in response to the expansion of the outbreak of COVID-19. The effective periods and coverage of each DSE are available in Supplementary material. During the research period of this study, the second, third, and fourth DSE were in effect. Despite some studies suggest that they were not as effective in strengthened the regional containment of the infection risk as the first DSE (5, 38, 39), it’s deemed important to consider their impact. Furthermore, the study acknowledges the changes in the social environment and the utility loss experienced by residents due to the implementation of these measures, which are considered as part of the cost of COVID-19 prevention and control. Therefore, including these aspects as input indicators is considered reasonable.

Regarding the output indicator, the recovery rate is expected to reflect the effectiveness of the healthcare system and medical institutions in the treatment and management of COVID-19 patients. The denominator of this indicator reflects the combined pressure on the healthcare system from the pandemic itself and the demographic characteristics of the region, and the numerator reflects the actual output of the healthcare system under this pressure. It’s worth noting that in Japan, severe COVID-19 patients typically require hospitalization, while those with moderate and mild symptoms are usually advised to undergo accommodation-based or home-based care until they are no longer considered a public health threat. Consequently, the denominator, in fact, represents the total number of patients who achieved discharge or release from treatment during the given period. Furthermore, considering that the average number of days of hospitalization is approximately 10 (40), a lag of 10 days was set for the number of infected patients requiring inpatient care, since recovery at time point t should be attributed to infections and medical practices prior to time point t.

### 2.2 Moderating role of vaccination: OLS model, Tobit model, and SUE *t*-test

To explore the socioeconomic factors influencing LHSE scores assessed by the DEA, I employed pooled Tobit models for cross-sectional data in my study. Additionally, I utilized the pooled OLS model to ensure the robustness of my findings. Previous research often used Tobit models to identify the factors impacting LHSE scores, considering these scores as censored at 1 (15, 16, 41). However, a study by McDonald (42) demonstrated that LHSE scores are not generated through a censoring process, suggesting that Tobit estimates might be inconsistent. In contrast, OLS estimates are consistent. Therefore, to enhance the robustness of the research findings, I employ the OLS model in the main body of the article and subsequently conduct a robustness check using the Tobit model.

The LHSE determination models before and after vaccine introduction can be specified as Equations 2 and 3,respectively:

For Model 1 (pre-vaccine introduction):

(2) $$LHSE_{iw}=\alpha_{0}+\alpha_{1}X_{iw}+u_{iw},w=2,3$$

For Model 2 (post-vaccine introduction):

(3) $$LHSE_{iw}=\beta_{0}+\beta_{1}X_{iw}+v_{iw},w=4,5$$

In these models, i represents different DMUs, w represents different waves of the pandemic, $X_{iw}$ denotes socioeconomic factors. Considering the real-world scenario, it is assumed that $\mathrm{cov}\left(u_{iw},v_{iw}\right)\neq 0$ implying a correlation between the error terms in the two models.

This research characterizes socioeconomic factors from multiple angles, including the role of local authorities (43, 44), the stress on the healthcare system (41), demographic characteristics (14, 15, 41), and the viral characteristics. Specifically, the role of local authorities is represented by the financial index and the proportion of newly confirmed cases not linked to known transmission sources. A higher financial index indicates a surplus of financial resources. The unlinked proportion of newly confirmed cases reflects the local government’s ability to control the spread of the virus. Healthcare system stress is gauged by the occupancy rates of total available beds and beds reserved for severe cases. Demographic characteristics, such as population density and the percentage of residents over 65 years old, are considered key factors affecting LHSE scores. Additionally, I examined the impact of SARS-CoV-2 and its variants on the LHSE score, focusing on the severity rate (indicative of pathogenicity) and the positivity rate (indicative of infectiousness). You can find detailed definitions for each variable in Table 3. Furthermore, regional dummies were included in the model to investigate spatial changes in LHSE scores.

**Table 3:** Notation and definition of explanatory variables for Tobit and OLS.

	Explanatory variable		Additional descriptions
Demographic characteristics	Population density	Unit: people per 1,000 m^2^ of total area.
	Old ratio	The proportion of the population aged 65 and older.	Ability of local authority	Financial index	The average of the last three years of $\frac{\mathrm{basic}\;\mathrm{amount}\;\mathrm{of}\;\mathrm{financial}\;\mathrm{receipt}}{\mathrm{basic}\;\mathrm{financial}\;\mathrm{need}}$
	Unlinked proportion	Unlinked proportion of newly confirmed cases	Healthcare system stress	Bed rate	Occupancy rate of total secured bed
	Bed rate for severe	Occupancy rate of secured bed for severe cases	Characteristics of virus	Positive rate	Percentage of COVID-19 positives
	Severe rate	$\mathrm{Severe}\;\mathrm{rate}=\frac{\mathrm{Number}\;\mathrm{of}\;\mathrm{severe}\;\mathrm{cases}}{\mathrm{Number}\;\mathrm{of}\;\mathrm{the}\;\mathrm{newly}\;\mathrm{confirmed}\;\mathrm{cases}\;\mathrm{with}\;a\;10-\mathrm{day}\;\mathrm{lag}}$	Region disparities	Hokkaido	Equals 1 for DMU = Hokkaido
	Tohoku	Equals 1 for DMU = Aomori, Iwate, Miyagi, Akita, Yamagata, and Fukushima
	Kanto	Equals 1 for DMU = Ibaraki, Tochigi, Gunma, Saitama, Chiba, Tokyo, Kanagawa
	Chubu	Equals 1 for DMU = Niigata, Toyama, Ishikawa, Fukui, Yamanashi, Nagano, Gifu, Shizuoka, and Aichi
	Kinki	Equals 1 for DMU = Mie, Shiga, Kyoto, Osaka, Hyogo, Nara, and Wakayama
	Kyushu	Equals 1 for DMU = Fukuoka, Saga, Nagasaki, Kumamoto, Oita, Miyazaki, Kagoshima, and Okinawa

(1) Considering that the current year’s medical activity was actually more affected by the previous year’s financial situation, I introduced a lag period to financial index. I.e., assuming that LHSE in year t is affected by the financial index in year *t*−1.

(2) The population density, old ratio, and financial index are only available for annual data, so the data of these three variables for wave3 is defined across years as the weighted average of the 2019 data and 2020 data.

(3) Population density, the proportion of the population aged 65 and older, and financial index data are sourced from the Statistics Bureau of Japan, while all other data is derived from data provided by the Ministry of Health, Labour and Welfare of Japan.

I confirm the moderating effect of the vaccine by examining the differences in estimated coefficients between Model 1 and Model 2. For this purpose, I propose the null hypothesis $H_{0}:\alpha_{1}=\beta_{1}$ . Due to the assumption that $\mathrm{cov}\left(u_{iw},v_{iw}\right)\neq 0$ , the traditional Hausman test is not applicable. Therefore, I am using the SUE *t*-test as an alternative. The SUE *t*-test offered several advantages compared to the Houseman test. First, it allowed us to estimate the covariance of coefficients and the entire model. Second, it facilitated the estimation of the covariance matrix across models and the testing of whether common coefficients were significantly equal or not (45). It’s important to note that for the SUE command in Stata 16, the estimator should be initially estimated without cluster or robust options, although cluster and robust options are permissible when SUE returns results.

In addition, apart from the analysis based on all DMUs, I conducted a heterogeneity analysis based on the *per capita* bed capacity of each DMU. I used the average *per capita* bed capacity of all DMUs as a benchmark, identifying DMUs with lower *per capita* bed capacity as those with insufficient bed resources and DMUs with higher *per capita* bed capacity as those with sufficient bed resources. Then, I separately conducted regressions and analyzed the moderating effect of vaccines on both DMUs with insufficient bed resources and those with sufficient bed resources. It’s well-known that there is significant variation in *per capita* bed capacity among Japan’s prefectures. With increasing infection numbers, the issue of insufficient bed capacity in local hospitals has at times pushed the healthcare system to the brink of collapse. For example, during the fourth wave of the pandemic, Okinawa Prefecture had bed utilization rates of 67.84% and intensive care bed utilization rates of 66.33%. Given that a higher *per capita* bed capacity may provide local healthcare institutions with more resilience in the face of medical resource shortages caused by a major COVID-19 outbreak, as a supplement to the analysis of all DMUs, I conducted a heterogeneous analysis of DMUs with both higher and lower *per capita* bed capacities.

## 3 Results

### 3.1 The efficiency of healthcare system

Table 4 presents descriptive statistics for the input and output indicators. It is important to note that the number of vaccine doses administered is not included in the input indicators for LHSE estimates during the second and third waves of the pandemic. Additionally, since no region declared a DSG during the second wave of the pandemic, the input indicators for the LHSE estimates of the second wave do not include the DSG input. Moreover, Japan is typically considered to have 47 prefectures, i.e., DMUs. However, due to the unavailability of some ECSG data for a portion of Gunma Prefecture, the actual sample includes only 46 prefectures.

**Table 4:** Descriptive statistics of inputs and outputs.

	Wave 2			Wave 3		
	*N*	Mean	SD	*N*	Mean	SD
ECSG_MIS	46	938107.380	1104064.470	46	6541031.200	2659120.537
ECSG_BA	46	397081.650	305367.742	46	1529229.800	997050.751
ECSG_FD	46	98820.097	340399.623	46	755761.960	341609.188
ECSG_RCP	46	9693.825	20513.025	46	111151.830	81244.769
DSE	–	–	–	46	0.083	0.158
PCR	46	9828.920	6628.207	46	40900.036	19461.983
Vaccine	–	–	-	–	–	–

	Wave 4			Wave 5		
	*N*	Mean	SD	*N*	Mean	SD
ECSG_MIS	46	726842.500	1117745.258	46	5947831.100	3041608.713
ECSG_BA	46	344237.890	290138.578	46	1528321.400	947704.983
ECSG_FD	46	39690.806	216108.418	46	305048.660	356603.132
ECSG_RCP	46	1238.880	5184.250	46	21755.318	29455.925
DSE	46	0.089	0.179	46	0.122	0.151
PCR	46	58762.116	28634.123	46	63814.889	31419.429
Vaccine	46	30.134	2.958	46	45.063	3.455

Table 5 reports the LHSE scores estimated by the DEA, with DMUs considered efficient receiving a score of 1 and marked in green. It’s essential to understand that DMUs encounter different external conditions and possess varying technological capabilities during different stages of the pandemic (different waves). Therefore, the LHSE estimates for each wave of the pandemic are cross-sectional. In theory, the absolute values of LHSE for different DMUs in different waves are not directly comparable. However, based on the technical efficiency measurement method introduced by Farrell (32), the LHSE estimated using the DEA method represents the gap between the evaluated DMU and perfectly efficient DMUs. For instance, in the 4th and 5th waves of the pandemic, Hokkaido has LHSE values of 0.859 and 0.842, respectively. I cannot conclude that Hokkaido’s technical efficiency was higher in the 4th wave compared to the 5th wave, as I cannot assume that the external environment and technological capabilities were identical in both waves. However, it is evident that in the 4th wave, Hokkaido had a smaller gap with DMUs considered efficient compared to the 5th wave. Furthermore, the arithmetic mean of LHSE for each wave of the pandemic does not represent the average level of technical efficiency across DMUs in the time series. Instead, it signifies the overall gap between the inefficient DMUs and the efficient DMU within the same wave.

**Table 5:** Healthcare efficiency scores of the DMUs.

DMUs	Unavailable vaccination		Available vaccination	
	Wave 2	Wave 3	Wave 4	Wave 5
Hokkaido	0.248	0.319	0.392	0.334
Aomori	0.355	0.760	1.000	0.562
Iwate	1.000	0.505	1.000	1.000
Miyagi	0.315	1.000	0.785	0.369
Akita	0.443	1.000	1.000	0.654
Yamagata	0.175	1.000	0.704	0.919
Fukushima	0.207	0.380	0.675	0.455
Ibaraki	0.078	0.500	0.829	0.559
Tochigi	0.293	0.816	0.766	0.720
Saitama	0.118	0.460	0.584	0.657
Chiba	0.113	0.584	1.000	0.583
Tokyo	0.041	1.000	0.303	0.329
Kanagawa	0.104	0.286	1.000	0.511
Niigata	0.313	1.000	1.000	1.000
Toyama	0.159	0.544	0.847	0.591
Ishikawa	0.091	0.390	0.598	0.539
Fukui	0.227	1.000	0.838	1.000
Yamanashi	0.133	0.827	0.833	1.000
Nagano	1.000	0.549	0.827	0.735
Gifu	0.116	1.000	0.685	0.634
Shizuoka	0.570	0.709	1.000	1.000
Aichi	0.400	0.509	1.000	1.000
Mie	0.216	0.719	1.000	0.395
Shiga	0.276	1.000	1.000	1.000
Kyoto	0.097	0.388	0.574	0.408
Osaka	0.077	0.328	0.194	0.392
Hyogo	0.317	0.366	0.299	0.562
Nara	0.152	0.385	0.802	1.000
Wakayama	0.228	0.685	0.800	0.642
Tottori	0.118	1.000	0.316	1.000
Shimane	0.172	1.000	1.000	1.000
Okayama	0.115	0.658	0.683	0.342
Hiroshima	0.492	1.000	0.458	0.313
Yamaguchi	0.335	0.574	1.000	1.000
Tokushima	1.000	0.664	0.766	1.000
Kagawa	0.289	0.535	0.695	1.000
Ehime	1.000	1.000	1.000	1.000
Kochi	0.612	0.750	1.000	1.000
Fukuoka	0.264	0.362	0.471	1.000
Saga	0.384	0.626	0.945	0.350
Nagasaki	0.254	0.435	0.595	0.711
Kumamoto	0.360	0.439	1.000	1.000
Oita	0.142	0.677	0.638	0.778
Miyazaki	0.790	0.675	0.852	1.000
Kagoshima	0.371	0.804	0.868	0.611
Okinawa	0.134	0.419	0.334	0.251
Proportion of efficient DMUs	0.087	0.261	0.326	0.391
Mean	0.319	0.666	0.760	0.715

Concerning the distribution of LHSE scores across prefectures, several noteworthy findings have emerged. Firstly, during the 2nd to 5th waves of the pandemic, the lowest LHSE values were 0.041, 0.286, 0.194, and 0.251, respectively observed in Tokyo, Kanagawa, Osaka, and Okinawa. Secondly, only one DMU, Ehime Prefecture, has managed to maintain perfect efficiency across all four waves of the pandemic, indicating that most DMUs do not possess inherent advantages that make it easy for them to consistently achieve efficiency. Thirdly, overall, the proportion of efficient DMUs increased steadily from 8.7% during the 2nd wave of the pandemic to 39.1% in the 5th wave. Meanwhile, the average LHSE rose from 0.319 in the second wave to 0.760 in the fourth wave, then experienced a slight decline to 0.715 during the 5th wave of the pandemic.

### 3.2 The moderating role of vaccination

Table 6 provides the descriptive statistics for the socioeconomic factors influencing LHSE. In.

**Table 6:** Descriptive statistics of explanatory variables for Tobit and OLS.

	Wave 2			Wave 3		
	*N*	Mean	SD	*N*	Mean	SD
Population density	46	0.665	1.235	46	0.663	1.234
Old ratio	46	0.307	0.032	46	0.262	0.058
Financial index	46	0.519	0.193	46	0.520	0.190
Unlinked proportion	46	0.328	0.160	46	0.320	0.123
Bed rate	46	0.123	0.107	46	0.240	0.133
Bed rate for severe	46	0.053	0.078	46	0.131	0.132
Positive rate	46	0.028	0.017	46	0.042	0.021
Severe rate	46	0.374	0.749	46	0.300	0.160

	Wave 4			Wave 5		
	*N*	Mean	SD	*N*	Mean	SD
Population density	46	0.662	1.233	46	0.662	1.233
Old ratio	46	0.311	0.032	46	0.311	0.032
Financial index	46	0.521	0.189	46	0.521	0.189
Unlinked proportion	46	0.373	0.128	46	0.367	0.146
Bed rate	46	0.285	0.111	46	0.183	0.054
Bed rate for severe	46	0.148	0.136	46	0.098	0.089
Positive rate	46	0.043	0.022	46	0.070	0.038
Severe rate	46	0.250	0.233	46	0.100	0.068

Table 7, these factors are used as independent variables in an OLS regression, with LHSE estimated through DEA as the dependent variable. This identifies the contributions of various socioeconomic factors to LHSE before and after vaccine deployment. Table 8 presents SUE *t*-tests based on the estimated coefficients from the pre-and post-vaccine deployment. These tests are used to demonstrate the moderating effect of vaccines on the socioeconomic inequality of LHSE. In order to present the analysis results more clearly, I have consolidated the simplified outcomes from Tables 7, 8 into Table 9. Furthermore, for a robustness test, Tobit model were used instead of OLS models, and the same procedures were conducted. The results are provided in Supplementary material. By contrasting the outcomes of the main regression and robustness tests, I observed consistency regarding the moderating effect of vaccinations on the association between socioeconomic factors, regional disparities, and the distribution of LHSE. This indicates a relative robustness in the findings of this study.

**Table 7:** Results of pooled OLS regression.

Variables	All DMUs			DMUs with insufficient bed resources			DMUs with sufficient bed resources		
	Full sample	Unavailable vaccination	Available vaccination	Full sample	Unavailable vaccination	Available vaccination	Full sample	Unavailable vaccination	Available vaccination
Population density	0.008	−0.014	−0.013	−0.002	−0.010	−0.033	0.308	0.161	0.692**
	(0.284)	(−0.306)	(−0.394)	(−0.060)	(−0.198)	(−0.960)	(1.264)	(0.496)	(2.236)
Old ratio	−0.037	−1.647**	2.347	−1.695	−0.780	0.665	0.029	−1.620**	7.612*
	(−0.070)	(−2.363)	(1.470)	(−1.162)	(−0.363)	(0.211)	(0.049)	(−2.135)	(2.025)
Financial index	−0.481**	−0.403	0.143	−0.797**	−0.741	0.274	−1.426**	−0.648	−0.345
	(−2.052)	(−1.161)	(0.471)	(−2.276)	(−1.292)	(0.508)	(−2.583)	(−0.915)	(−0.463)
Unlinked proportion	−0.386*	−0.558**	−0.348	−0.360	−0.466	−0.261	−0.297	−0.278	−0.776
	(−1.868)	(−1.993)	(−1.174)	(−1.376)	(−1.212)	(−0.731)	(−0.843)	(−0.677)	(−1.388)
Bed rate	0.993***	−0.002	0.850*	1.229***	0.532	1.897**	0.073	−0.189	−0.862
	(3.028)	(−0.004)	(1.813)	(2.884)	(0.673)	(2.220)	(0.132)	(−0.200)	(−1.271)
Bed rate for severe	−0.898**	0.453	−1.198**	−0.425	0.762	−1.812**	0.666	−1.359	1.293
	(−2.228)	(0.688)	(−2.515)	(−0.836)	(0.908)	(−2.471)	(0.600)	(−0.679)	(1.034)
Positive rate	3.756***	2.884	2.244**	3.323***	2.523	1.232	5.116***	3.291	1.785
	(4.491)	(1.249)	(2.421)	(3.288)	(0.734)	(0.997)	(3.486)	(0.983)	(1.107)
Severe rate	−0.088*	−0.107*	0.119	−0.113**	−0.109*	−0.403	0.321	0.824**	0.335
	(−1.698)	(−1.845)	(0.805)	(−2.109)	(−1.747)	(−0.760)	(1.588)	(2.285)	(1.354)
Tohoku	0.056	0.142	0.006	0.048	−0.187	0.301*	0.144	0.400**	−0.295
	(0.765)	(1.260)	(0.074)	(0.305)	(−0.700)	(1.748)	(0.987)	(2.069)	(−1.365)
Kanto	0.004	−0.024	0.117	−0.023	−0.330	0.364**	–	–	–
	(0.046)	(−0.168)	(1.092)	(−0.150)	(−1.259)	(2.177)			
Chubu	0.056	0.077	0.093	0.113	−0.165	0.356**	−0.100	−0.232	0.017
	(0.816)	(0.722)	(1.169)	(0.748)	(−0.647)	(2.141)	(−0.851)	(−1.392)	(0.117)
Kinki	−0.176**	−0.161	−0.067	−0.257	−0.532**	0.230	−0.373*	−0.553**	−0.414
	(−2.332)	(−1.436)	(−0.719)	(−1.621)	(−2.047)	(1.211)	(−1.924)	(−2.271)	(−1.502)
Kyushu	−0.170**	−0.225**	−0.015	−0.945***	−1.154***	0.094	−0.110	−0.027	−0.010
	(−2.511)	(−2.279)	(−0.190)	(−3.386)	(−2.769)	(0.239)	(−1.475)	(−0.264)	(−0.100)
Constant	0.783***	1.289***	−0.152	1.404**	1.386*	−0.033	0.961***	1.063***	−1.580
	(3.790)	(5.037)	(−0.238)	(2.548)	(1.832)	(−0.025)	(3.372)	(2.896)	(−1.117)
R-squared	0.266	0.287	0.347	0.422	0.426	0.592	0.319	0.532	0.411
Observations	184	92	92	104	52	52	80	40	40
Number of DMU	46	46	46	26	26	26	20	20	20

*t*-statistics in parentheses. *** *p* < 0.01, ** *p* < 0.05, * *p* < 0.1.

**Table 8:** Results of SUE *t*-test based on OLS regression.

Variables	All DMUs			DMUs with insufficient bed resources			DMUs with sufficient bed resources		
	Diff.	Chi2	Prob>chi2	Diff.	Chi2	Prob>chi2	Diff.	Chi2	Prob>chi2
Population density	0.001	0.00	0.980	−0.023	0.28	0.599	0.531	2.49	0.114
Old ratio	3.994**	6.25	0.012	1.445	0.31	0.580	9.232**	5.38	0.020
Financial index	0.546	1.67	0.196	1.015	2.49	0.114	0.303	0.10	0.758
Unlinked proportion	0.210	0.30	0.584	0.205	0.17	0.684	−0.498	0.87	0.351
Bed rate	0.852	1.47	0.226	1.365	2.16	0.142	−0.673	0.51	0.474
Bed rate for severe	−1.651*	3.51	0.061	−2.574**	6.04	0.014	2.652*	2.81	0.094
Positive rate	−0.640	0.09	0.764	−1.291	0.14	0.705	−1.506	0.24	0.627
Severe rate	0.226*	3.03	0.082	−0.294	0.56	0.456	−0.489	1.60	0.207
Tohoku	−0.136	0.95	0.329	0.488***	7.66	0.006	−0.695***	7.64	0.006
Kanto	0.141	1.00	0.317	0.694***	21.14	0.000	Omitted	Omitted	Omitted
Chubu	0.016	0.01	0.903	0.521***	10.53	0.001	0.249	1.61	0.205
Kinki	0.094	0.49	0.484	0.762***	16.86	0.000	0.139	0.30	0.584
Kyushu	0.210*	3.50	0.061	1.248***	10.88	0.001	0.017	0.02	0.879

(1) The SUE *t*-test statistics (Chi2) are estimated based on robust standard errors, *** *p* < 0.01, ** *p* < 0.05, * *p* < 0.1.

(2) “Diff.” indicates the difference in value between estimated coefficients with vaccination and those without vaccination.

**Table 9:** Summary of pooled OLS and SUE *t*-test results.

	Explanatory variables	All DMUs			DMUs with insufficient bed resources			DMUs with sufficient bed resources		
		UV	AV	Changes in magnitude	UV	AV	Changes in magnitude	UV	AV	Changes in magnitude
Demographic characteristics	Population density	−	−	↓	−	−	↑	+	+**	↑
	Old ratio	−**	+	↑**	−	+	↓	−**	+*	↑**
Ability of local authority	Financial index	−	+	↓	−	+	↓	−	−	↓
	Unlinked proportion	−**	−	↓	−	−	↓	−	−	↑
Healthcare system stress	Bed rate	−	+*	↑	+	+**	↑	−	−	↑
	Bed rate for severe	+	−**	↑*	+	−**	↑**	−	+	↓*
Characteristics of virus	Positive rate	+	+**	↓	+	+	↓	+	+	↓
	Severe rate	−*	+	↑*	−*	−	↑	+**	+	↓
Region disparities	Tohoku	+	+	↓	−	+*	↑***	+**	−	↓***
	Kanto	−	+	↑	−	+**	↑***	Omitted	Omitted	Omitted
	Chubu	+	+	↑	−	+**	↑***	−	+	↓
	Kinki	−	−	↓	−**	+	↓***	−**	−	↓
	Kyushu	−**	−	↓*	−***	+	↓***	−	−	↓

(1) “UV” and “AV” respectively represent “Unavailable Vaccination” and “Available Vaccination”.

(2) “+” and “−” indicate the positive and negative signs of the estimated coefficients for each variable in the pooled OLS regression. Subsequent “*” aligns with Table 7.

(3) “↑” and “↓” signify an increase or decrease in the absolute value of the estimated coefficients for each variable in the pooled OLS regression following the introduction of vaccine deployment. Subsequent “*” corresponds to Table 8.

#### 3.2.1 Demographic characteristics

I utilized population density and the proportion of population aged 65 and older to depict the demographic impact on LHSE. Initially, the impact of population density on LHSE was found to be significant only for DMUs with sufficient bed resources post-vaccination, with higher-density DMUs exhibiting higher LHSE scores. Secondly, concerning the proportion of population aged 65 and older, before vaccine deployment, a higher proportion led to lower LHSE. However, vaccination shifted this influence from negative to positive, although this effect could not be statistically proven. Nevertheless, the moderating effect of vaccination on the relationship between the proportion of individuals aged 65 and older and LHSE was significant. Additionally, in regions with sufficient bed resources, the moderating effect of vaccines was more pronounced. Furthermore, even after vaccination, the positive correlation between the proportion of population aged 65 and older and LHSE remained statistically significant at a 90% confidence level in DMUs with sufficient bed resources.

#### 3.2.2 Ability of local authority

Overall, the financial index of DMUs was negatively associated with LHSE, although this relationship wasn’t individually confirmed in the pre-and post-vaccination datasets. Moreover, the unlinked proportion was negatively correlated with LHSE pre-vaccination. The vaccination’s impact on the moderating effect of local authorities’ ability on LHSE was not significant, regardless of the full sample, DMUs with abundant bed resources, or those with scarce resources.

#### 3.2.3 Healthcare system stress

Overall, the overall bed occupancy rate is positively correlated with LHSE, while the intensive care bed occupancy rate is negatively correlated. For DMUs with insufficient bed resources, post-vaccination, LHSE became more dependent on both these occupancy rates. However, the vaccine’s usage does not significantly moderate the relationship between total bed occupancy rate and LHSE. Nevertheless, the vaccine’s usage significantly strengthened the contribution of intensive care bed occupancy rates to LHSE for DMUs with limited bed resources, amplifying it by a factor of 2.37 and transforming their positive correlation into a negative one.

#### 3.2.4 Characteristics of virus

Regardless of whether DMUs had sufficient or insufficient bed resources, there was a significant positive correlation between Positive rate and LHSE. Although severe rate was generally negatively correlated with LHSE, in regions with more available bed resources, higher severe case rates led to higher LHSE pre-vaccination. Additionally, there was no evidence indicating that the advent of vaccines regulated the relationship between positive rate, severe rate, and LHSE.

#### 3.2.5 Region disparities

Based on the estimated coefficients of regional dummy variables, the introduction of vaccines primarily regulated regional disparities in LHSE in areas with insufficient bed resources. Specifically, for DMUs with scarce bed resources, the vaccine’s moderating effect on regional disparities was significant. After vaccine deployment, the Tohoku, Kanto, and Chubu regions significantly outperformed others in LHSE. However, the Kinki and Kyushu regions were significantly disadvantaged pre-vaccination, and post-vaccination, this difference was no longer significant. For DMUs with abundant bed resources, the introduction of vaccines was found to weaken the relative advantage of the Tohoku region in LHSE.

## 4 Discussion

LHSE estimation results offer evidence to evaluate the overall performance of all DMUs. Firstly, except for Ehime Prefecture, no other prefectures managed to sustain efficient across the 4 waves of the pandemic. This indicates that achieving high LHSE solely through exogenous factors like socioeconomics and luck is challenging for most DMUs. Moreover, the rising trend in average LHSE and the percentage of efficient DMUs among all DMUs indicates a continuous narrowing of the gap between DMUs and the efficiency frontier during each pandemic wave, suggesting an overall reduction in efficiency disparities among DMUs. Compared to the vaccine-unavailable 2nd and 3rd waves, there was a notable improvement in the overall performance of DMUs during the 4th and 5th waves post-vaccination. Furthermore, analyses using OLS and SUE *t*-tests on regional dummies indicate that the usage of vaccines indeed impacted the uneven distribution of LHSE, primarily focusing on regions with insufficient bed resources. After vaccine deployment in regions with insufficient bed resources, the Tohoku, Kanto, and Chubu regions transformed from LHSE underperformers to overperformers, while the relative disadvantage of the Kinki and Kyushu regions pre-vaccination became insignificant post-vaccination.

The analysis of demographic characteristics reveals that before vaccine deployment, DMUs with a higher proportion of older adult population susceptible to COVID-19 faced greater resistance in improving LHSE. However, post-vaccination, DMUs with a higher proportion of older adult individuals, particularly those with ample bed resources, found it easier to improve LHSE. This might be due to Japan’s early policy of prioritizing vaccination for the older adult. Even after vaccinating the general population, the vaccination rate among the older adult remained higher than other age groups. Thus, improving LHSE became relatively easier in DMUs with a larger proportion of older adult individuals.

Regarding the analysis of local government ability and LHSE, vaccine deployment did not mitigate LHSE disparities caused by differences in local government capabilities. Nevertheless, the negative correlation between the financial coefficient and LHSE indicates that DMUs with better financial conditions found it harder to improve LHSE. Strong financial conditions usually reflect a better economy and complex demographics. Therefore, regions with better financial conditions faced more challenges in accurately controlling the pandemic’s direction during a sudden public health crisis, leading to lower input–output efficiency.

The analysis on virus characteristics’ relationship with LHSE shows that the impact of virus characteristics on LHSE is not significantly regulated by vaccine usage.

The relationship between healthcare system stress and LHSE, and the moderating effect of vaccine deployment on this relationship, were proven. High total secured bed occupancy rates led to higher LHSE, representing a combination of two causal chains: higher bed occupancy rates indicate a severe COVID-19 situation, increased demand for medical resources, and stress on the healthcare system, risking a decrease in LHSE scores. Simultaneously, it implies that existing medical resources are actively used in medical activities, preventing a surge in mortality rates, hence boosting LHSE scores. Moreover, Inoue (1) suggests that Japan effectively controlled the pandemic early on by coordinating optimal use of hospital beds at the community level, irrespective of low or high occupancy rates. Additionally, post-vaccination, LHSE improvement became more reliant on reducing the occupancy rate of beds for severe cases, especially for DMUs with inadequate bed resources. This suggests that, in the post-vaccine era, focusing on measures like ensuring supply of intensive care beds and treatment for severe patients could be crucial for improving LHSE.

Additionally, strengthening the review and oversight of the ECSG funds would contribute to an overall enhancement of LHSE. A report published by the Japan’s audit watchdog indicates that 66 ECSG projects where funds were wasted or misused, totaling 10.23 billion yen from the 2019 fiscal year to the 2021 fiscal year. This included overpayments of around 5.5 billion yen to 32 hospitals with aside beds for treating COVID-19 patients. The ECSG is designed to pay for the cost of beds reserved specifically for COVID-19 patients that were never used or for bed costs that could not be used due to the need for space to treat COVID-19 patients. However, all 32 hospitals applied for funds, even during periods when beds were occupied. Another case is that four hospitals classified regular beds as advanced care units and received an extra payment totaling about 3.1 billion yen. A senior official from the Board Audit said “Many hospitals had an inadequate understanding of the program. The prefectural governments handling the payments were also lax in their initial evaluation.” A senior official from the Board Audit attributed part of this problem to the lax initial assessments conducted by the prefectural governments (46).

## 5 Conclusion

This study examined whether the use of vaccines during Japan’s COVID-19 waves two through five mitigated inequalities in the spatial distribution and socioeconomic factors of LHSE. The findings indicate an overall reduction in LHSE disparities across regions due to vaccine utilization. However, this change varied concerning the availability of hospital bed resources in prefectures. In prefectures facing bed shortages, vaccine usage notably improved LHSE in most areas. Conversely, in prefectures with ample bed resources, vaccine deployment only significantly decreased the advantage of the Tohoku region, resulting in a more even overall performance among prefectures.

Nevertheless, the study’s outcomes did not provide evidence supporting vaccines as a leveling factor in reducing socioeconomic LHSE disparities. On one hand, vaccine deployment increased the contribution of the population aged 65 and above to LHSE, particularly accentuated in regions with sufficient bed resources. On the other hand, the improvement in LHSE in prefectures, especially those with insufficient bed resources, relied more on reducing the occupancy rate of secured beds for severe cases due to vaccine deployment.

These results suggest that policymakers and implementers, in the post-COVID-19 era with rising vaccine coverage, should prioritize bolstering and supplying medical resources conducive to treating severe cases, such as secured beds for severe cases, especially in regions with limited bed resources. Additionally, I posit that the positive impact of the older adult population on LHSE due to initial vaccine prioritization might change as vaccination coverage among younger demographics increases.

Given the frequent alterations in preventive policies, it’s impossible to encompass all potential influencing factors in the model determining medical efficiency. Consequently, any overlooked influencing factors would be included in the model’s residual term, leading to endogeneity issues. For instance, during the second wave of COVID-19, the “Go To Travel” tourism promotion policy implemented from July 22, 2020, to December 28, 2020, was proven to significantly increase infection rates (47, 48). Moreover, the hosting of the Tokyo Olympics and Paralympics exacerbated the pandemic’s spread (49, 50). Therefore, validation of research findings is imperative upon acquiring more data and statistical information related to COVID-19.

## Data availability statement

The original contributions presented in the study are included in the article/Supplementary material, further inquiries can be directed to the corresponding author.

## Author contributions

The author confirms being the sole contributor of this work and has approved it for publication.
