# Supplementary material for: Socioeconomic inequalities in healthcare system efficiency in Japan during COVID-19 pandemic: an analysis of the moderating role of vaccination
Source: Front Public Health. 2024 Mar 21;12:1170628. doi: 10.3389/fpubh.2024.1170628 (PMC10996399; doi:10.3389/fpubh.2024.1170628)
Supplement: Supplementary file 1 [file Data_Sheet_1.docx]

Supplementary Material

**Supplementary Figure 1.** The beginning and end of the declaration of the state of emergency

| DMUs | the first DSE | |  | the second DSE | |  | the third DSE | |  | the fourth DSE | |
| --- | --- | --- | --- | --- | --- | --- | --- | --- | --- | --- | --- |
|  | start | end |  | start | end |  | start | end |  | start | end |
| Hokkaido | 2020/4/16 | 2020/5/25 |  |  |  |  | 2021/5/16 | 2021/6/20 |  | 2021/8/25 | 2021/9/30 |
| Aomori | 2020/4/16 | 2020/5/14 |  |  |  |  |  |  |  |  |  |
| Iwate | 2020/4/16 | 2020/5/14 |  |  |  |  |  |  |  |  |  |
| Miyagi | 2020/4/16 | 2020/5/14 |  |  |  |  |  |  |  | 2021/8/25 | 2021/9/13 |
| Akita | 2020/4/16 | 2020/5/14 |  |  |  |  |  |  |  |  |  |
| Yamagata | 2020/4/16 | 2020/5/14 |  |  |  |  |  |  |  |  |  |
| Fukushima | 2020/4/16 | 2020/5/14 |  |  |  |  |  |  |  |  |  |
| Ibaraki | 2020/4/16 | 2020/5/14 |  |  |  |  |  |  |  | 2021/8/20 | 2021/9/30 |
| Tochigi | 2020/4/16 | 2020/5/14 |  | 2021/1/14 | 2021/2/7 |  |  |  |  | 2021/8/20 | 2021/9/30 |
| Gunma | 2020/4/16 | 2020/5/14 |  |  |  |  |  |  |  | 2021/8/20 | 2021/9/30 |
| Saitama | 2020/4/7 | 2020/5/25 |  | 2021/1/8 | 2021/3/21 |  |  |  |  | 2021/8/2 | 2021/9/30 |
| Chiba | 2020/4/7 | 2020/5/25 |  | 2021/1/8 | 2021/3/21 |  |  |  |  | 2021/8/2 | 2021/9/30 |
| Tokyo | 2020/4/7 | 2020/5/25 |  | 2021/1/8 | 2021/3/21 |  | 2021/4/25 | 2021/6/20 |  | 2021/7/12 | 2021/9/30 |
| Kanagawa | 2020/4/7 | 2020/5/25 |  | 2021/1/8 | 2021/3/21 |  |  |  |  | 2021/8/2 | 2021/9/30 |
| Niigata | 2020/4/16 | 2020/5/14 |  |  |  |  |  |  |  |  |  |
| Toyama | 2020/4/16 | 2020/5/14 |  |  |  |  |  |  |  |  |  |
| Ishikawa | 2020/4/16 | 2020/5/14 |  |  |  |  |  |  |  |  |  |
| Fukui | 2020/4/16 | 2020/5/14 |  |  |  |  |  |  |  |  |  |
| Yamanashi | 2020/4/16 | 2020/5/14 |  |  |  |  |  |  |  |  |  |
| Nagano | 2020/4/16 | 2020/5/14 |  |  |  |  |  |  |  |  |  |
| Gifu | 2020/4/16 | 2020/5/14 |  | 2021/1/14 | 2021/2/26 |  |  |  |  | 2021/8/25 | 2021/9/30 |
| Shizuoka | 2020/4/16 | 2020/5/14 |  |  |  |  |  |  |  | 2021/8/20 | 2021/9/30 |
| Aichi | 2020/4/16 | 2020/5/14 |  | 2021/1/14 | 2021/2/26 |  | 2021/5/12 | 2021/6/20 |  | 2021/8/25 | 2021/9/30 |
| Mie | 2020/4/16 | 2020/5/14 |  |  |  |  |  |  |  | 2021/8/25 | 2021/9/30 |
| Shiga | 2020/4/16 | 2020/5/14 |  |  |  |  |  |  |  | 2021/8/25 | 2021/9/30 |
| Kyoto | 2020/4/16 | 2020/5/21 |  | 2021/1/14 | 2021/2/26 |  | 2021/4/25 | 2021/6/20 |  | 2021/8/20 | 2021/9/30 |
| Osaka | 2020/4/7 | 2020/5/21 |  | 2021/1/14 | 2021/2/26 |  | 2021/4/25 | 2021/6/20 |  | 2021/8/2 | 2021/9/30 |
| Hyogo | 2020/4/7 | 2020/5/21 |  | 2021/1/14 | 2021/2/26 |  | 2021/4/25 | 2021/6/20 |  | 2021/8/20 | 2021/9/30 |
| Nara | 2020/4/16 | 2020/5/14 |  |  |  |  |  |  |  |  |  |
| Wakayama | 2020/4/16 | 2020/5/14 |  |  |  |  |  |  |  |  |  |
| Tottori | 2020/4/16 | 2020/5/14 |  |  |  |  |  |  |  |  |  |
| Shimane | 2020/4/16 | 2020/5/14 |  |  |  |  |  |  |  |  |  |
| Okayama | 2020/4/16 | 2020/5/14 |  |  |  |  | 2021/5/16 | 2021/6/20 |  | 2021/8/25 | 2021/9/13 |
| Hiroshima | 2020/4/16 | 2020/5/14 |  |  |  |  | 2021/5/16 | 2021/6/20 |  | 2021/8/25 | 2021/9/30 |
| Yamaguchi | 2020/4/16 | 2020/5/14 |  |  |  |  |  |  |  |  |  |
| Tokushima | 2020/4/16 | 2020/5/14 |  |  |  |  |  |  |  |  |  |
| Kagawa | 2020/4/16 | 2020/5/14 |  |  |  |  |  |  |  |  |  |
| Ehime | 2020/4/16 | 2020/5/14 |  |  |  |  |  |  |  |  |  |
| Kochi | 2020/4/16 | 2020/5/14 |  |  |  |  |  |  |  |  |  |
| Fukuoka | 2020/4/7 | 2020/5/14 |  | 2021/1/14 | 2021/2/26 |  | 2021/5/12 | 2021/6/20 |  | 2021/8/20 | 2021/9/30 |
| Saga | 2020/4/16 | 2020/5/14 |  |  |  |  |  |  |  |  |  |
| Nagasaki | 2020/4/16 | 2020/5/14 |  |  |  |  |  |  |  |  |  |
| Kumamoto | 2020/4/16 | 2020/5/14 |  |  |  |  |  |  |  |  |  |
| Oita | 2020/4/16 | 2020/5/14 |  |  |  |  |  |  |  |  |  |
| Miyazaki | 2020/4/16 | 2020/5/14 |  |  |  |  |  |  |  |  |  |
| Kagoshima | 2020/4/16 | 2020/5/14 |  |  |  |  |  |  |  |  |  |
| Okinawa | 2020/4/16 | 2020/5/14 |  |  |  |  | 2021/5/23 | - |  | - | 2021/9/30 |

**Supplementary Figure 2.** Results of Pooled Tobit regression

| VARIABLES | All DMUs | | |  | DMUs with insufficient bed resources | | |  | DMUs with sufficient bed resources | | |
| --- | --- | --- | --- | --- | --- | --- | --- | --- | --- | --- | --- |
|  | Full  sample | Unavailable  vaccination | Available  vaccination |  | Full  sample | Unavailable  vaccination | Available  vaccination |  | Full  sample | Unavailable  vaccination | Available  vaccination |
| Population density | 0.024 | -0.010 | -0.001 |  | 0.012 | -0.001 | -0.035 |  | 0.493 | 0.226 | 1.038** |
|  | (0.657) | (-0.197) | (-0.012) |  | (0.314) | (-0.010) | (-0.829) |  | (1.663) | (0.734) | (2.556) |
| Old ratio | 0.062 | -1.851** | 2.740 |  | -2.487 | -1.508 | 0.432 |  | 0.250 | -1.725** | 10.795** |
|  | (0.092) | (-2.390) | (1.217) |  | (-1.335) | (-0.640) | (0.104) |  | (0.340) | (-2.370) | (2.122) |
| Financial index | -0.590* | -0.465 | 0.188 |  | -0.984** | -0.955 | 0.448 |  | -1.934*** | -0.762 | -0.686 |
|  | (-1.940) | (-1.213) | (0.427) |  | (-2.236) | (-1.580) | (0.635) |  | (-2.779) | (-1.124) | (-0.647) |
| Unlinked proportion | -0.479* | -0.618** | -0.562 |  | -0.448 | -0.542 | -0.358 |  | -0.350 | -0.296 | -1.092 |
|  | (-1.803) | (-1.998) | (-1.319) |  | (-1.391) | (-1.343) | (-0.786) |  | (-0.800) | (-0.749) | (-1.322) |
| Bed rate | 1.166*** | -0.102 | 1.137* |  | 1.487*** | 0.533 | 2.593** |  | -0.262 | -0.356 | -1.410 |
|  | (2.771) | (-0.158) | (1.731) |  | (2.815) | (0.645) | (2.402) |  | (-0.380) | (-0.388) | (-1.517) |
| Bed rate for severe | -1.181** | 0.583 | -1.577** |  | -0.717 | 0.894 | -2.470** |  | 1.108 | -1.322 | 2.086 |
|  | (-2.280) | (0.801) | (-2.345) |  | (-1.137) | (1.005) | (-2.647) |  | (0.795) | (-0.698) | (1.169) |
| Positive rate | 5.131*** | 3.312 | 3.635*** |  | 4.188*** | 2.891 | 1.590 |  | 7.905*** | 3.920 | 4.210* |
|  | (4.602) | (1.271) | (2.703) |  | (3.303) | (0.811) | (1.041) |  | (3.865) | (1.162) | (1.788) |
| Severe rate | -0.102 | -0.123* | 0.096 |  | -0.127* | -0.123* | -0.703 |  | 0.424* | 0.915** | 0.432 |
|  | (-1.573) | (-1.927) | (0.476) |  | (-1.955) | (-1.904) | (-1.065) |  | (1.736) | (2.671) | (1.400) |
| Tohoku | 0.049 | 0.166 | -0.030 |  | 0.058 | -0.242 | 0.365* |  | 0.161 | 0.462** | -0.498 |
|  | (0.512) | (1.307) | (-0.256) |  | (0.303) | (-0.853) | (1.767) |  | (0.861) | (2.374) | (-1.686) |
| Kanto | -0.055 | -0.042 | 0.034 |  | -0.056 | -0.410 | 0.381* |  | - | - | - |
|  | (-0.459) | (-0.276) | (0.227) |  | (-0.300) | (-1.473) | (1.906) |  |  |  |  |
| Chubu | 0.044 | 0.080 | 0.057 |  | 0.144 | -0.215 | 0.406* |  | -0.156 | -0.261 | -0.041 |
|  | (0.488) | (0.675) | (0.500) |  | (0.785) | (-0.794) | (2.019) |  | (-1.112) | (-1.657) | (-0.231) |
| Kinki | -0.248** | -0.194 | -0.150 |  | -0.299 | -0.641** | 0.288 |  | -0.538** | -0.631** | -0.657* |
|  | (-2.542) | (-1.569) | (-1.132) |  | (-1.547) | (-2.289) | (1.258) |  | (-2.297) | (-2.712) | (-1.889) |
| Kyushu | -0.256*** | -0.283** | -0.106 |  | -1.108*** | -1.430*** | 0.146 |  | -0.203** | -0.054 | -0.139 |
|  | (-2.922) | (-2.582) | (-0.915) |  | (-3.139) | (-2.999) | (0.291) |  | (-2.161) | (-0.555) | (-1.068) |
| Constant | 0.860*** | 1.433*** | -0.197 |  | 1.775** | 1.829** | -0.070 |  | 1.075*** | 1.136*** | -2.401 |
|  | (3.245) | (5.023) | (-0.219) |  | (2.492) | (2.123) | (-0.041) |  | (3.079) | (3.231) | (-1.255) |
|  |  |  |  |  |  |  |  |  |  |  |  |
| Observations | 184 | 92 | 92 |  | 104 | 52 | 52 |  | 80 | 40 | 40 |
| Number of DMU | 46 | 46 | 46 |  | 26 | 26 | 26 |  | 20 | 20 | 20 |

Supplementary Figure 3. Results of SUE t-test based on Tobit regression

| VARIABLES | All DMUs | | |  | DMUs with insufficient bed resources | | |  | DMUs with sufficient bed resources | | |
| --- | --- | --- | --- | --- | --- | --- | --- | --- | --- | --- | --- |
|  | Diff. | Chi2 | Prob>chi2 |  | Diff. | Chi2 | Prob>chi2 |  | Diff. | Chi2 | Prob>chi2 |
| Population density | 0.009 | 0.03 | 0.865 |  | -0.034 | 0.39 | 0.532 |  | 0.812* | 3.23 | 0.072 |
| Old ratio | 4.591** | 3.98 | 0.046 |  | 1.940 | 0.18 | 0.670 |  | 12.520** | 4.29 | 0.038 |
| Financial index | 0.653 | 1.22 | 0.270 |  | 1.403 | 2.17 | 0.141 |  | 0.076 | 0.00 | 0.956 |
| Unlinked proportion | 0.056 | 0.01 | 0.919 |  | 0.184 | 0.07 | 0.793 |  | -0.796 | 0.80 | 0.372 |
| Bed rate | 1.239 | 1.72 | 0.189 |  | 2.060 | 2.55 | 0.110 |  | -1.054 | 0.60 | 0.437 |
| Bed rate for severe | -2.160* | 3.67 | 0.055 |  | -3.364** | 5.57 | 0.018 |  | 3.408 | 2.52 | 0.112 |
| Positive rate | 0.323 | 0.01 | 0.912 |  | -1.301 | 0.10 | 0.752 |  | 0.290 | 0.00 | 0.950 |
| Severe rate | 0.219 | 1.47 | 0.226 |  | -0.580 | 1.00 | 0.318 |  | -0.483 | 1.09 | 0.296 |
| Tohoku | -0.196 | 1.01 | 0.314 |  | 0.607** | 5.86 | 0.016 |  | -0.960** | 5.60 | 0.018 |
| Kanto | 0.076 | 0.17 | 0.678 |  | 0.791*** | 13.23 | 0.000 |  | - | - | - |
| Chubu | -0.023 | 0.02 | 0.899 |  | 0.621*** | 6.93 | 0.009 |  | 0.220 | 0.80 | 0.371 |
| Kinki | 0.044 | 0.05 | 0.815 |  | 0.929*** | 12.11 | 0.001 |  | -0.026 | 0.01 | 0.940 |
| Kyushu | 0.177 | 1.22 | 0.270 |  | 1.576 | 6.71 | 0.010 |  | -0.085 | 0.30 | 0.587 |
